# Classifying Major Depressive Disorder Using Multimodal MRI Data: A Personalized Federated Algorithm

**DOI:** 10.3390/brainsci15101081

**Published:** 2025-10-06

**Authors:** Zhipeng Fan, Jingrui Xu, Jianpo Su, Dewen Hu

**Affiliations:** College of Intelligence Science and Technology, National University of Defense Technology, Changsha 410073, China; fzp_0097@nudt.edu.cn (Z.F.); xujingrui@nudt.edu.cn (J.X.); sujianpo10@nudt.edu.cn (J.S.)

**Keywords:** major depressive disorder, multimodal MRI data, personalized federated learning, gradient matching, model contrastive optimization

## Abstract

Background: Neuroimaging-based diagnostic approaches are of critical importance for the accurate diagnosis and treatment of major depressive disorder (MDD). However, multisite neuroimaging data often exhibit substantial heterogeneity in terms of scanner protocols and population characteristics. Moreover, concerns over data ownership, security, and privacy make raw MRI datasets from multiple sites inaccessible, posing significant challenges to the development of robust diagnostic models. Federated learning (FL) offers a privacy-preserving solution to facilitate collaborative model training across sites without sharing raw data. Methods: In this study, we propose the personalized Federated Gradient Matching and Contrastive Optimization (pF-GMCO) algorithm to address domain shift and support scalable MDD classification using multimodal MRI. Our method incorporates gradient matching based on cosine similarity to weight contributions from different sites adaptively, contrastive learning to promote client-specific model optimization, and multimodal compact bilinear (MCB) pooling to effectively integrate structural MRI (sMRI) and functional MRI (fMRI) features. Results and Conclusions: Evaluated on the Rest-Meta-MDD dataset with 2293 subjects from 23 sites, pF-GMCO achieved accuracy of 79.07%, demonstrating superior performance and interpretability. This work provides an effective and privacy-aware framework for multisite MDD diagnosis using federated learning.

## 1. Introduction

Major depressive disorder (MDD) remains one of the most prevalent neuropsychiatric conditions globally, characterized by persistent low mood and significant cognitive deficits [1]. With over 264 million individuals affected worldwide [2,3], MDD contributes significantly to functional impairment in daily and occupational contexts and represents a leading cause of disability [4]. In total, it accounts for nearly one million suicide-related deaths annually. Current diagnostic protocols rely predominantly on clinician judgment and patient self-reports, contributing to substantial underdiagnosis, particularly in low- and middle-income countries, where an estimated 76–85% of cases go without proper treatment [5]. These challenges highlight the urgent need for objective, biomarker-driven diagnostic tools to enable early intervention and alleviate pressures on mental healthcare systems. Neuroimaging-based classifiers have shown considerable potential in providing such tools, offering a scalable complement to clinical assessment.

Multimodal magnetic resonance imaging (MRI) has emerged as a key modality for identifying neurobiological alterations in MDD. Structural MRI (sMRI) reveals consistent abnormalities in cortical and subcortical morphology across multisite cohorts [6,7], while functional MRI (fMRI)—including both resting-state and task-based paradigms—has demonstrated dysregulation in large-scale neural networks [8,9,10,11,12]. These findings suggest that integrating sMRI and fMRI biomarkers could significantly enhance the accuracy and generalizability of automated diagnostic systems.

A major obstacle in developing such systems is the scarcity of single-site datasets sufficient for training robust models. While the centralized aggregation of multisite data is a common solution [13,14,15], it raises serious ethical and legal concerns regarding data privacy [16], particularly given the sensitive nature of neuroimaging data [17]. Federated learning (FL) provides a promising alternative by enabling decentralized model training without sharing raw data [18,19,20]. Among FL algorithms, federated averaging (FedAvg) is widely adopted for its efficiency [21], and preliminary applications to brain fMRI have demonstrated feasibility [22]. As a privacy-preserving deep learning paradigm, federated learning aims to build a model or system that can utilize multisite information without raw data transfer.

However, domain shift may occur due to non-biological variability that can be attributed to differences in scanner manufacturers, non-standardized imaging acquisition protocols, and other intrinsic factors across different sites, resulting in both domain-invariant and domain-specific features across sites [23,24,25]. There are different optimization directions for unique global models in different sites [26]. A unique global model, such as one trained via standard FedAvg, is trained primarily based on global information and fails to account for these domain-specific characteristics, often leading to suboptimal performance. In contrast, personalized FL models allow for site-specific adaptation while still leveraging shared knowledge across sites [27]. Our pF-GMCO explicitly aligns local and global representations through gradient matching and contrastive learning, thereby achieving a better trade-off between generalization and personalization. Thus, we prefer to establish the optimal pFL model rather than a unique global one.

To overcome these challenges, we propose a personalized federated learning algorithm, named Personalized Federated Gradient Matching and Contrastive Optimization (pF-GMCO), designed for multisite MDD classification using multimodal MRI. Our approach incorporates two novel technical components: (1) a gradient matching mechanism that adaptively weights contributions from different sites based on distribution similarity, effectively mitigating domain shifts, and (2) a model contrastive loss that promotes personalized model optimization tailored to each site’s data characteristics. Furthermore, we integrate multimodal compact bilinear pooling (MCB) [28] to capture high-order interactions between sMRI-derived morphological features and fMRI-derived representations (ReHo and ALFF), significantly enhancing feature integration and the discriminative power.

We evaluated our method on the Rest-Meta-MDD consortium dataset, comprising 2293 subjects from 23 sites. Compared to existing FL strategies and fusion methods, our method achieved state-of-the-art performance, with average classification accuracy of 79.07%, outperforming existing FL approaches and alternative multimodal fusion strategies, alongside favorable interpretability and robustness. The contributions of this work are as follows:

1. We propose pF-GMCO, a personalized federated learning algorithm that combines adaptive gradient matching with contrastive optimization to learn site-specific models while leveraging cross-site knowledge in a privacy-preserving manner.
2. We introduce an MCB-based fusion module for integrating sMRI and fMRI features, significantly improving the classification performance compared to unimodal and conventional fusion approaches.
3. We demonstrate the clinical applicability and superior performance of our framework through extensive multisite experiments, with visualization results highlighting biologically plausible regions such as the default mode network and frontoparietal network, consistent with the established MDD pathophysiology.

## 2. Materials and Methods

### 2.1. Data and Preprocessing

Multimodal MRI data from the REST-Mate-MDD project were included in this study, consisting of multimodal MRI data from MDD patients and matched healthy controls (HCs) from 25 imaging sites http://rfmri.org/REST-meta-MDD, (accessed on 1 January 2020). We used the data of 2293 subjects from 23 sites, including 1028 MDD patients and 1225 matched healthy controls (sites S4 and S19 were removed, as they exhibited some data overlap with other sites). Notably, the multimodal MRI data in the REST-meta-MDD project are publicly available, eliminating the need for ethical approval in our study. Considering the limited scale of some private datasets (such as #S5, #S12, etc.), it is difficult to optimize local models effectively. We randomly divided the 23 sites into five federated union clients, ensuring a similar range of subjects within each client. The details of the federated clients are shown in Table 1. Specifically, the collected subjects were matched in terms of gender, age, and education. The patients were diagnosed as having MDD based on the ICD10 or DSM-IV. The subject information and the imaging acquisition parameters of each site can be seen on the project website.

In this study, we use the T1-weighted brain sMRI data and resting-state fMRI data for experiments. Specifically, the sMRI data cover the 3D space and are preprocessed using the CAT-12.8 toolbox (fil.ion.ucl.ac.uk/spm/software/). Then, considering that the rs-fMRI data cover the 4D space and are not aligned with the 3D feature space of the sMRI data, we further calculate the low-frequency oscillations (ALFF) and regional homogeneity (ReHo) from the preprocessed resting-state fMRI data. Specifically, the ALFF and ReHo metrics are widely adopted in resting-state fMRI studies to capture localized neural activity and synchronization, which are frequently disrupted in MDD. Specifically, ALFF quantifies the intensity of spontaneous low-frequency oscillations (0.01–0.1 Hz), reflecting baseline neural activity levels [7,29], while ReHo measures the temporal similarity of BOLD signals within a local neighborhood, serving as an indicator of regional functional coherence [30]. Previous studies have demonstrated aberrant ALFF and ReHo patterns in MDD patients, underscoring their value as functional biomarkers of the disorder.

The projection of 4D rs-fMRI into a 3D space for ALFF and ReHo extraction provides two key advantages for multimodal fusion: (1) it preserves the critical spatial patterns of brain function while ensuring dimensional compatibility with sMRI features, which is a prerequisite for unified model design; (2) it establishes consistent latent granularity that allows structural and functional information to complement each other effectively, ultimately leading to more computationally efficient and discriminative joint representations for the diagnosis of MDD.

### 2.2. Method Overview

**Figure 1:**
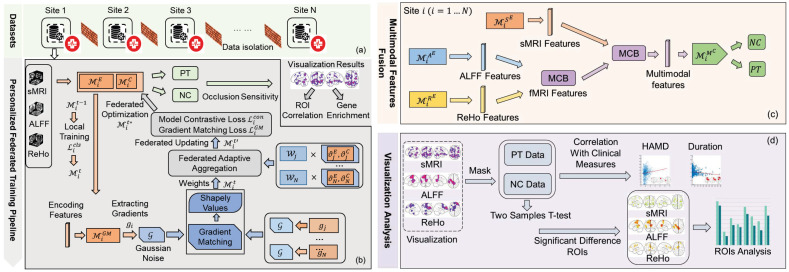
An overview of our study. (**a**) represents the isolation of private MRI datasets in multiple medical sites; (**b**) is the pF-GMCO pipeline used to train pFL models; (**c**) is the fusion strategy for multimodal features; (**d**) describes the pipeline of visualization and analysis.

The proposed pFL algorithm mainly includes two updating steps for the training of personalized federated models. Specifically, we propose a novel adaptive aggregating mechanism, based on calculating the federated gradient matching loss. Then, we introduce gradient matching and the model contrastive loss for regularization to further alleviate domain shifts and enable personalized optimization. The pipeline of pF-GMCO can be seen in Figure 1b, and the details are shown in Algorithm 1.
**Algorithm 1** pFL -GMCO Algorithm**Require:** Private datasets ${\left\{D_{i}=\left(x_{i}^{S},x_{i}^{A},x_{i}^{R}\right),Y_{i}\right\}}_{i=1}^{N}$ in multiple sites, pFL models ${\left\{M_{i}=M_{i}^{E},M_{i}^{C},M_{i}^{GM}\right\}}_{i=1}^{N}$, number of epochs *L*, local learning rate α, federated learning rate η, variance $\sigma^{2}$ of Gaussian noise, local learning rate α, federated learning rate η.**Ensure:** Optimal weights of pFL model $M_{i}$ in site $F_{i}$;  1:**for** t=1:L **do**  2:    Local updating:  3:    **for** i=1:N **do**  4:        Compute local classification loss: $L_{i}^{cls}$  5:        Update pFL models locally: $\theta_{i}^{t}\leftarrow \theta_{i}^{t-1}-\alpha \nabla_{\theta_{i}^{t-1}}L_{i}^{cls}$  6:    **end for**  7:    Federated adaptive aggregation:  8:    **for** i=1:N **do**  9:        Compute gradient matching loss: $L_{i}^{GM}$10:        Compute the adaptive aggregating weights: $\lambda_{i,j}$11:        Federated aggregation: $\theta_{i}^{t^{\prime }}\leftarrow \theta_{i}^{t}+\zeta \sum_{j=1(j\neq i)}^{N}\lambda_{i,j}G\left(\theta_{j}^{t}\right)$12:    **end for**13:    Federated optimization:14:    **for** i=1:N **do**15:        Compute gradient matching loss: $L_{i}^{Fed-GM}$16:        Compute model contrastive loss: $L_{i}^{con}$17:        Compute classification loss: $L_{i}^{con}$18:        Compute federated optimization loss: $L_{i}^{Fed}=L_{i}^{Fed-cls}+\beta L_{i}^{Fed-GM}+\gamma L_{i}^{con}$19:        Update personalized federated parameters: $\theta_{i}^{t*}\leftarrow \theta_{i}^{t^{\prime }}-\eta \nabla_{\theta_{i}^{t^{\prime }}}L_{i}^{Fed}$20:    **end for**21:    Update multimodal classifier: $\theta_{i}^{MLP^{t}*}\leftarrow \theta_{i}^{MLP^{t}}-\alpha \nabla_{\theta_{i}^{MLP^{t}}}L_{CE}\left(M_{i}^{MLP}\left(z_{MCB}\right),Y_{i}\right)$22:**end for**23:Return the pFL model $M_{i}$ for each federated site.

Then, we introduce the MCB strategy to fuse mulitmodal features encoded from sMRI and fMRI data, making full use of structural and functional information. It aims to utilize the advantages of different types of MRI data and improve the performance of MDD classification in each MRI dataset.

We next provide the details of our proposed pFL algorithm. Specifically, there are *N* medical sites in the federation. The private dataset in federated site $F_{i}$ can be denoted as $D_{i}=\left\{X_{i},Y_{i}\right\}$. $X_{i}$ denotes the brain MRI data, containing the sMRI data $x_{i}^{S}\in R^{H\times W\times L}$, ALFF data $x_{i}^{A}\in R^{\frac{H}{2}\times \frac{W}{2}\times \frac{L}{2}}$, and ReHo data $x_{i}^{R}\in R^{\frac{H}{2}\times \frac{W}{2}\times \frac{L}{2}}$. $Y_{i}\in \left[0,1\right]$ represents the diagnostic label of each subject, where 0 indicates normal controls and 1 indicates MDD patients, respectively. Our objective is to train a pFL model $M_{i}=M_{i}^{S},M_{i}^{A},M_{i}^{R}$ for site $F_{i}$ to improve the performance of MDD classification in $D_{i}$ without transferring raw data from other federated sites $F_{j}\left(j\neq i\right)$. Significantly, we only introduce the details of the pFL models’ federated training process using sMRI data here; the pFL models using ALFF and ReHo data share the same training process. To simplify the expressions, we use $M_{i}$ here to represent the pFL models for sMRI data, containing two main modules: (1) encoder $M_{i}^{E}$ and classifier $M_{i}^{C}$ and (2) gradient matching module $M_{i}^{GM}$.

A federated encryption mechanism is essential for sharing gradient information across sites [31], and differential privacy encryption [32] has a relatively low computational load and communication consumption. Specifically, it provides a privacy-preserving bound to ensure that private or sensitive information is not exposed in the shared information. Inspired by a previous study, we found that adding noise, such as Gaussian noise, to federated information is an effective way to realize differential privacy and limit the granularity of the information. Thus, this provides a good privacy guarantee for each federated site. Specifically, we construct the Gaussian noise generator G(·) for each site, which can be defined as

(1) $$G\left(P\right)=P+N\left(0,\sigma^{2}\right),$$

where *P* represents the federated information shared from the federated site $F_{j}$, such as gradients or model parameters. $N(0,\sigma^{2})$ represents the Gaussian noise for privacy preservation (mean value is 0 and variance value is $\sigma^{2}$). Hereby, differential privacy of a specific privacy-preserving bound can be guaranteed across sites by linking the parameter $\sigma^{2}$ in this random encrypted mechanism. A larger $\sigma^{2}$ will blur the shared gradients more and can even be harmful for model training, so the balance between the degree of privacy preservation and model performance can be adjusted via the parameter $\sigma^{2}$.

### 2.3. Federated Adaptive Aggregation Based on Gradient Matching

Similarly to the implementation of the federated training algorithm [22], we split the updating process into local training and federated optimization. Specifically, we set $M_{i}^{0}$ as the initial model for site $F_{i}$. In iteration *t*, the model $M_{i}^{t}$ is updated using local dataset $D_{i}$ by calculating the loss in classifying diagnosis labels. We use the cross-entropy loss function $L_{CE}$ and the classification loss $L_{i}^{cls}$, which can be formulated as

(2) $$L_{i}^{cls}=E_{\left\{x_{i}^{S},Y_{i}\right\}∼D_{i}}\left[L_{CE}\left(M_{i}^{C^{t-1}}\left(M_{i}^{E^{t-1}}\left(x_{i}^{S}\right)\right),Y_{i}\right)\right].$$

Then, the parameters $\theta_{i}^{t}$ of model $M_{i}^{t}$ would be updated locally,

(3) $$\theta_{i}^{t}=\theta_{i}^{t-1}-\eta \nabla_{\theta_{i}^{t-1}}L_{i}^{cls},$$

where η is the learning rate. Then, model $M_{i}^{t-1}$ would be updated to $M_{i}^{t}$ and be ready for federated information aggregation after learning local knowledge.

We extract gradient information from the local training process of each site for federated adaptive aggregation [33]. Specifically, the gradient matching module $M_{i}^{GM}$ in site $F_{i}$ is encrypted and shared with the other sites $F_{j}\left(j\neq i\right)$ to calculate and aggregate the encrypted gradient $g_{j}^{t}$. Previous studies have shown that different features may have greater homogeneity when their gradients have a similar distribution. The core idea of the gradient matching mechanism is to maximize the similarity between gradients calculated by inputting different features with the same model parameters. As for site $F_{i}$, we use the encoding features of MRI data $f_{i}^{t}=M_{i}^{E^{t}}\left(x_{i}^{S}\right)$ as the input of the gradient matching model $M_{i}^{GM}$, which can be formulated as follows:

(4) $$\begin{array}{cc} g_{i}^{t} & =E_{\left\{x_{i}^{S},Y_{i}\right\}∼D_{i}}\left[\nabla_{\theta_{i}^{GM^{t}}}L_{CE}\left(M_{i}^{GM^{t}}\left(f_{i}^{t}\right),Y_{i}\right)\right],\\ g_{j}^{t} & =E_{\left\{x_{j}^{S},Y_{j}\right\}∼D_{j}}\left[\nabla_{G(\theta_{i}^{GM^{t}})}L_{CE}\left(M_{i}^{GM^{t}}\left(f_{j}^{t}\right),Y_{j}\right)\right],\\ L_{i,j}^{GM} & =1-\frac{{g_{i}^{t}}^{⊤}G\left(g_{j}^{t}\right)}{{\left||g_{i}^{t}|\right|}_{2}{\left||G(g_{j}^{t})|\right|}_{2}},\\ L_{i}^{GM} & =\sum_{j=1(j\neq i)}^{N}L_{i,j}^{GM}. \end{array}$$

Considering that the gradient matching loss can be seen as the similarity of the data distribution among the other sites $F_{j}\left(j\neq i\right)$ and local site $F_{i}$, we define the adaptive aggregating weights $\lambda_{i,j}^{t}$ for federated information $\theta_{j}^{t}$ in iteration *t*, which can be formulated as follows:

(5) $$\lambda_{i,j}=\frac{L_{i,j}^{GM}}{L_{i}^{GM}}.$$

Then, the encrypted local parameters from the federation can be weighted adaptively according to the gradient matching loss. The model would be updated by aggregating federated information,

(6) $$\theta_{i}^{t^{\prime }}=\theta_{i}^{t}+\zeta \sum_{j=1(j\neq i)}^{N}\lambda_{i,j}G\left(\theta_{j}^{t}\right),$$

where ζ is the regularization term for the parameters in federated aggregation. Thus, the specified aggregated weights can utilize more information from federations with a similar data distribution, thus alleviating the effects of domain shifts.

### 2.4. Federated Model Contrastive Optimization

The purpose of our federated algorithm is to train a federated personalized model for each site, which can enable better performance during MDD classification in local private datasets. Thus, the model should be updated towards site-specific optimization. Although federated adaptive aggregation can utilize global information from cross-site MRI datasets, the aggregated model may deviate from the local optimum and need to be corrected for site-specific personalization. Thus, we further propose personalized optimization based on the combination of the model contrastive loss and gradient matching loss.

We construct the model contrastive loss $L_{con}$ to measure the similarity in the features encoded by different models [34]. Specifically, we extract the encoding features of different models using the same MRI data $x_{i}^{S}$, including the federated features $f_{fed}=M_{i}^{E^{t^{\prime }}}\left(x_{i}^{S}\right)$, local features $f_{local}=M_{i}^{E^{t}}\left(x_{i}^{S}\right)$, and previous features $f_{pre}=M_{i}^{E^{t-1}}\left(x_{i}^{S}\right)$ encoded by the federated model updated in the last iteration t−1. The contrastive loss is based on calculating the cosine similarity $cos\left(a,b\right)=\frac{a^{⊤}b}{{\left||a|\right|}_{2}{\left||b|\right|}_{2}}$ between different encoding features. It can be formulated as

(7) $$L_{i}^{con}=E_{x_{i}^{S}∼D_{i}}\left[-log\frac{exp[cos\left(f_{fed},f_{local}\right)/\tau ]}{exp\left[1-cos\left(f_{fed},f_{local}\right)/\tau \right]+exp\left[1-cos\left(f_{fed},f_{pre}\right)/\tau \right]}\right],$$

where τ denotes the relevant parameters. Then, the contrastive loss would be minimized to ensure personalized optimization while aggregating federated information from the other sites.

We also maintain the gradient matching loss to further constrain the updating direction of the personalized federated model, which can prevent the pFL model from forgetting the global information. According to Equation (4), the gradient matching loss in federated optimization can be formulated as

(8) $$\begin{array}{cc} g_{i}^{t^{\prime }} & =E_{\left\{x_{i}^{S},Y_{i}\right\}∼D_{i}}\left[\nabla_{\theta_{i}^{GM^{t^{\prime }}}}L_{CE}\left(M_{i}^{GM^{t^{\prime }}}\left(f_{i}^{t^{\prime }}\right),Y_{i}\right)\right],\\ g_{j}^{t^{\prime }} & =E_{\left\{x_{j}^{S},Y_{j}\right\}∼D_{j}}\left[\nabla_{G(\theta_{i}^{GM^{t^{\prime }}})}L_{CE}\left(M_{i}^{GM^{t^{\prime }}}\left(f_{j}^{t^{\prime }}\right),Y_{j}\right)\right],\\ L_{i}^{Fed-GM} & =\sum_{j=1(j\neq i)}^{N}\left[1-\frac{{g_{i}^{t^{\prime }}}^{⊤}G\left(g_{j}^{t^{\prime }}\right)}{\left|\right|g_{i}^{t^{\prime }}{\left|\right|}_{2}{\left||G(g_{j}^{t^{\prime }})|\right|}_{2}}\right]. \end{array}$$

Then, the classification loss $L_{i}^{Fed-cls}$ for classifying the diagnosis labels can be formulated as

(9) $$L_{i}^{Fed-cls}=E_{\left(x_{i}^{S},Y_{i}\right)∼D_{i}}\left[L_{CE}\left(M_{i}^{C^{t^{\prime }}}\left(M_{i}^{E^{t^{\prime }}}\left(x_{i}^{S}\right)\right),Y_{i}\right)\right].$$

Finally, we build the federated optimized objective function and the personalized federated model $M_{i}^{*}$ with parameters $\theta_{i}^{*}=\left\{\theta_{i}^{E*},\theta_{i}^{C*},\theta_{i}^{GM*}\right\}$, which can be formulated as

(10) $$\begin{array}{cc} L_{i}^{Fed} & =L_{i}^{Fed-cls}+\beta L_{i}^{Fed-GM}+\gamma L_{i}^{con},\\ \theta_{i}^{*} & =\theta_{i}^{t^{\prime }}-\eta L_{i}^{Fed}, \end{array}$$

where β and γ denote the learnable parameters to scale and shift the federated loss value.

### 2.5. Fusion Strategy for Multimodal MRI Data

To extract and utilize more information to improve the performance of MDD diagnostic classification, we introduce a fusion strategy for multimodal MRI data. Considering the different feature spaces between 3D sMRI data and 4D rs-fMRI data, we calculated the ALFF and ReHo metrics based on the 4D rs-fMRI data to convert the functional features into 3D space. Inspired by previous studies, we use the MCB pooling method to combine and fuse multimodal features encoded from different MRI data. Notably, standard bilinear pooling computes the outer product of two feature vectors, resulting in a high-dimensional representation that captures all multiplicative interactions. To avoid the high computational cost, MCB uses count sketch based on a linear projection encoding layer and fast Fourier transform (FFT) for convolution. Thus, the MCB for different modality features can be formulated as

(11) $$\mathrm{MCB}\left(a,b\right)=F^{-1}\left(F(\Phi (a))⊙F(\Phi (b))\right),$$

where *F* denotes the FFT operation and $F^{-1}$ denotes the inverse FFT, while ⊙ is element-wise multiplication. Then, the vector $z_{MCB}=\mathrm{MCB}\left(a,b\right)$ is a compact representation capturing the multiplicative interactions between features *a* and *b*, which can fuse the different modality features effectively.

The fusion strategy can be seen in Figure 1c. Firstly, we use the single-modal MRI data to train a pFL model for each federated site. Then, we fuse the features encoded from the ALFF data and ReHo data for complete rs-fMRI representations using MCB pooling. After this, the rs-fMRI representations would be fused with features encoded from sMRI data to combine the structural and functional features. Thus, the fused multimodal MRI representation is $z_{MCB}=\mathrm{MCB}\left(M_{i}^{S}\left(x_{i}^{S}\right),\mathrm{MCB}\left(M_{i}^{A}\left(x_{i}^{A}\right),M_{i}^{R}\left(x_{i}^{R}\right)\right)\right)$. Then, we further construct a multilayer perceptron (MLP) network $M_{i}^{MLP}$ as a multimodal classifier for MDD diagnostic classification in site $F_{i}$. The parameters $\theta_{i}^{MLP}$ are updated while freezing the parameters of single-modality models $\theta_{i}^{S},\theta_{i}^{A},\theta_{i}^{R}$, which can be optimized as follows:

(12) $$\theta_{i}^{MLP^{t}}=\theta_{i}^{MLP^{t-1}}-\alpha \nabla_{\theta_{i}^{MLP^{t-1}}}L_{CE}\left(M_{i}^{MLP}\left(z_{MCB}\right),Y_{i}\right).$$

Thus, our method can not only train pFL models for single-modal MRI data but also can utilize and combine structural and functional features encoded from multimodal MRI data to further improve the performance of MDD diagnosis.

## 3. Results

### 3.1. Implementation Details

The models are trained on a Ubuntu 18.04.1 server with two eight-core Intel E5 2609 1.7GHz processors and four NVIDIA GTX-V100 graphical processing units. The codes are written in the Python 3.7.1 and Pytorch 1.13.1 frameworks [35]. Each model in our framework is trained for 80 epochs in total, and the batch size is set to 8. We use the AdamW optimizer [36] with weight decay of 0.95. The initial learning rate α is set to 0.01, and the federated learning rate η is set to 0.001. The federated aggregation process was performed once at the end of each epoch. The variance $\sigma^{2}$ of Gaussian noise is set to 0.001, according to the results of the control analysis presented in the next section. To avoid the additional influence caused by the network architecture, we use 3D-ResNet-10 as the backbone for model training to compare the performance of different learning algorithms.

Then, the number of the encoding features from the sMRI data, ALFF, and ReHo is 1024 based on 3D-ResNet-10. Notably, the MCB pooling strategy used for the fusion of multimodal features would not change the number of latent features. Thus, the fused multimodal feature number remains 1024. To simplify the structure of the MLP module, we use three-layer fully connected layers. Specifically, the size of the first layer corresponds to the dimensions of the encoded features from the fusion of MRI data. The second layer’s size is set to 128. As all tasks involve binary classification for MDD diagnosis, the size of the output layer is 2.

The experiments were carried out using a five-fold cross-validation strategy. Four folds were used for model training and the remaining one for testing. We used the average accuracy (ACC) and area under the curve (AUC) during cross-validation to evaluate the classification performance.

### 3.2. Comparison with SOTA Federated Learning Methods

We first train a local MDD identification model using single-modal MRI data for each client as the baseline comparison. We use 3D-ResNet-10 as the backbone for subsequent experiments. To evaluate the performance of our federated learning algorithm, we compare it against several widely used FL methods: centralized learning (aggregating all site data into a unified training set), FedAvg [22] (aggregating model parameters from each site to optimize a unique global model), FedProx [37] (an improved version of FedAvg with a regularization term), and FedALA [38] (a personalized FL method with adaptive local aggregation) and our proposed pF-GMCO.

**Figure 2:**
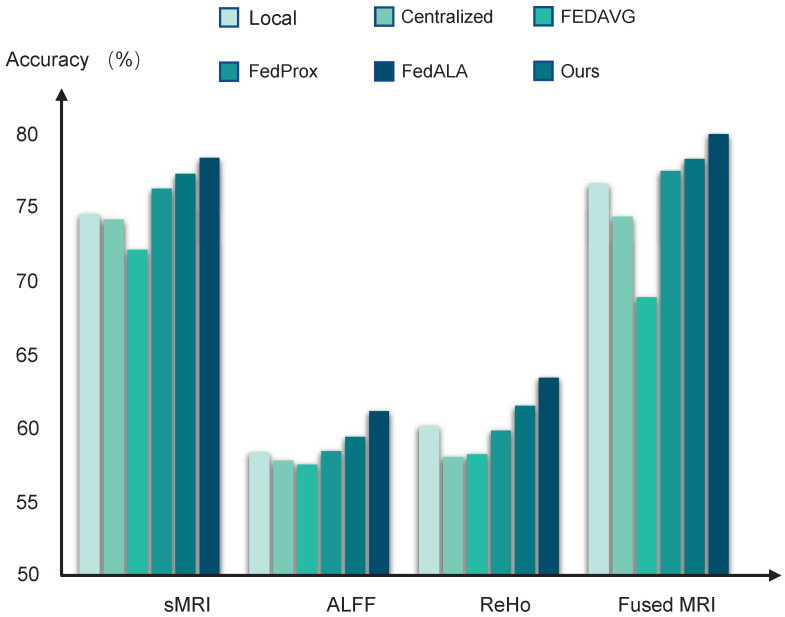
The average AUC values of different training algorithms in MDD diagnostic classification.

The performance of the diagnostic models trained by different learning algorithms can be found in Table 2 and Figure 2. The local models achieved average accuracy of 74.55 ± 0.31%, 58.23 ± 0.39%, and 60.10 ± 0.42%, based on using sMRI data, ALFF data, and ReHo data, respectively. Compared to these local baselines, the centralized learning approach does not yield significant improvements, likely due to the adverse effects of domain shifts when aggregating multisite data directly. Furthermore, models trained using FedAvg perform even more poorly than the local models, suggesting that the naive averaging of heterogeneous model parameters can impair federated model performance.

In contrast, federated methods, which are designed to mitigate domain shift, such as FedProx with the addition of a regularized constraint and FedALA with adaptive aggregation, show improved performance. Among all compared methods, our pF-GMCO algorithm achieves state-of-the-art results in MDD classification across multisite MRI data. The ACCs of our method across all federated sites reach up to 77.19 ± 0.25%, 61.43 ± 0.29%, and 64.17 ± 0.35% when using sRMI data, ALFF data, and ReHo data, respectively, demonstrating its effectiveness in leveraging distributed data while preserving privacy and adapting to personalized characteristics.

### 3.3. Multimodal MRI Data Fusion

Then, we fuse the multimodal MRI data based on the MCB pooling method for MDD diagnostic classification. The results can be seen in Table 2 and Figure 2. It can be observed that the models trained by fusing multimodal MRI data significantly improved the performance compared to models trained by single-modality MRI data. Specifically, the averaged ACCs of the pFL models trained by our proposed method reached up to 79.07%, meeting the 80% clinically relevant accuracy threshold.

### 3.4. Control Analysis

To comprehensively evaluate the effectiveness of the proposed pF-GMCO algorithm under various conditions, we conducted a series of controlled experiments focusing on three critical variables: adaptive aggregation weighting, the privacy–utility trade-off, and robustness against dishonest federated participants.

**Figure 3:**
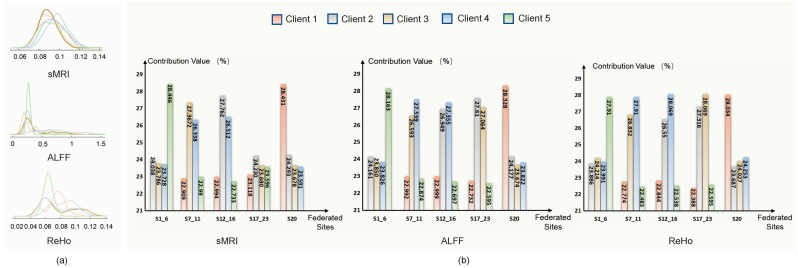
The average aggregated weights of federated information in each client. (**a**) shows the data distributions of different types of MRI data in each client; (**b**) shows the different contribution values evaluated by gradient matching in each client.

The adaptive aggregation strategy in pF-GMCO is designed to utilize domain-invariant information by quantifying the similarity of the data distribution across different clients. As illustrated in Figure 3, the assigned aggregation weights of each client are strongly correlated with the similarity. Specifically, pF-GMCO effectively prioritizes information from clients with higher similarity, enhancing the relevance and quality of federated updating. This demonstrates the capacity to integrate multiclient available knowledge dynamically and meaningfully.

**Figure 4:**
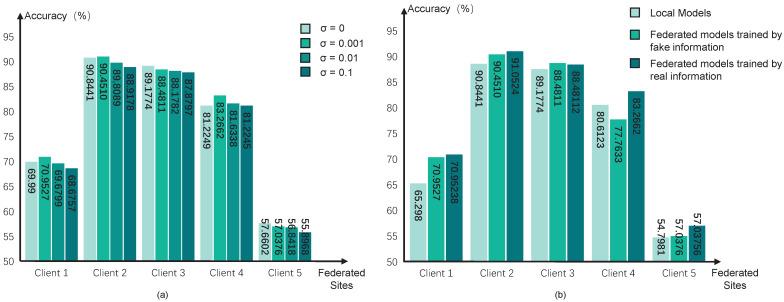
The control analysis of hyperparameters. (**a**) shows the performance of the pFL model with different Gaussian noise variance $\sigma^{2}$; (**b**) shows the performance of the pFL model trained with different degrees of fake federated information.

Then, we test different values of variance $\sigma^{2}$ in the Gaussian noise for encryption. A larger value of $\sigma^{2}$ means a higher degree of privacy preservation, but the optimization directions would be more unclear, which may reduce the performance of pFL models. We test the variance with four values, i.e., 0, 0.1, 0.01, and 0.001, and the performance can be seen in Figure 4a. The results indicate that the degree of encryption yields a trade-off between the performance of diagnostic classification and data privacy preservation. We choose $\sigma^{2}=0.001$ in our pFL algorithm.

Considering the dishonest participant sites in the federation, we further test the influence of using fake federated information in our pFL algorithm. We simulate scenarios in which all federated sites share the fake information for the federated training process, and the results can be seen in Figure 4b. Specifically, we generate random fake information in each site and share it with the other sites for federated updating. However, it can be observed that our pF-GMCO algorithm consistently sustained high performance across all federated clients, even when there were dishonest participant clients with fake information. This illustrates that our algorithm has the capacity to mitigate the impact of invalid federated information and maintain reliable performance even in non-ideal federation environments.

### 3.5. Ablation Study

To verify the effectiveness of our pF-GMCO algorithm, we conduct ablation studies using multiclient sMRI data for MDD diagnostic classification, and the results are shown in Table 3. It is shown that federated adaptive aggregation can utilize domain-invariant information from multiple sites based on the similarity of the data distributions, which can improve the performance of FL models. The FL models trained by the adaptive aggregation method outperform the FL models trained by FedAvg, which proves that considering domain shift in the FL training processes can improve the performance of models significantly. Moreover, federated optimization based on the constraints of gradient matching and model contrastive loss can further optimize FL models towards local personalization. When all personalized federated strategies are applied, the diagnostic classification abilities of the pFL models can be significantly enhanced.

### 3.6. Most Discriminative ROIs and Visualization Analysis

To identify the most discriminative regions of interest (ROIs) for the diagnostic classification of major depressive disorder (MDD), we construct occlusion sensitivity maps for different types of MRI data [39]. The maps are obtained by replacing volumetric patches within the 3D MRI feature maps with zero-value voxels of an equivalent size and evaluating the corresponding decrease in the prediction accuracy. A greater reduction in accuracy upon the occlusion of a specific ROI indicates higher discriminative power. Note that the ROI heat map is generated only based on the data-driven method, and we do not consider age, sex, the first episode or recurrence, and other clinical factors.

**Figure 5:**
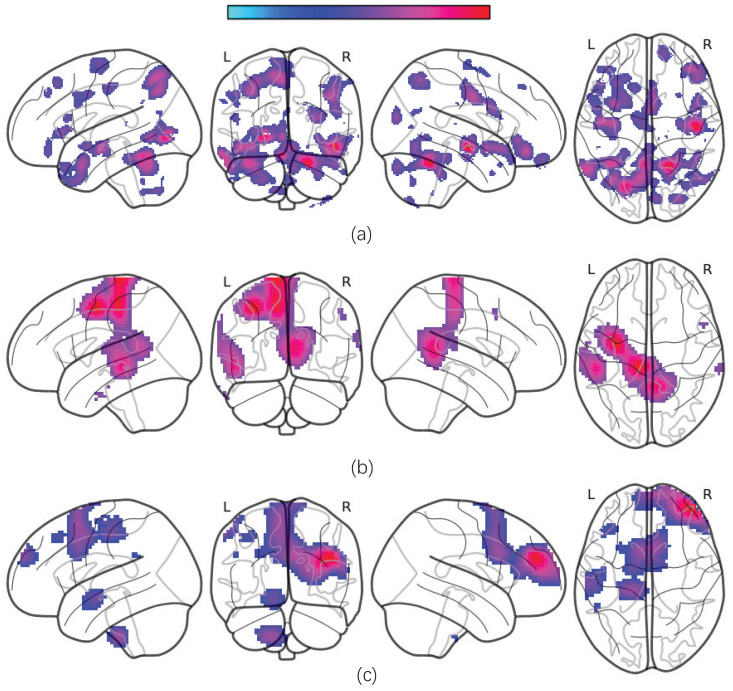
The visualization results for different types of MRI data. (**a**) sMRI occlusion sensitivity maps; (**b**) ALFF occlusion sensitivity maps; (**c**) ReHo occlusion sensitivity maps.

The average occlusion sensitivity maps for each MRI modality are presented in Figure 5.

Our results show that gray matter volumes (GMVs) are significantly reduced in several brain regions, including the temporal cortex, angular cortex, hippocampus, posterior cingulate cortex, orbitofrontal cortex, superior frontal cortex, superior parietal cortex, supplementary motor area (SMA), and certain cerebellar subregions. These alterations are consistent with previously reported disruptions in the cortico-limbic-cerebellar circuit in MDD patients [40,41]. Additionally, we observed significant decreases in the amplitudes of low-frequency fluctuations (ALFF) and regional homogeneity (ReHo) within these regions.

**Figure 6:**
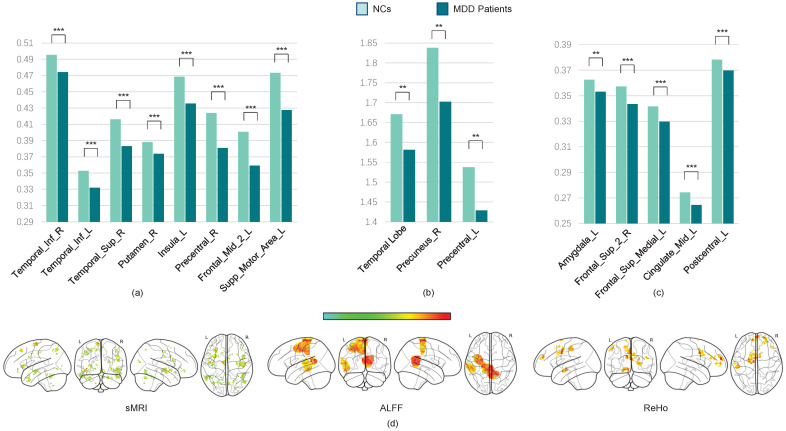
The most significant discriminative brain ROIs for MDD diagnostic classification. (**a**) The most significant ROIs in sMRI data; (**b**) the most significant ROIs in ALFF data; (**c**) the most significant ROIs in ReHo data; (**d**) the visualization results for the most significant discriminative ROIs in MRI data. The “**” indicate statistically significant differences between NCs and MDD that *p* value ≤ 0.05. “***” indicates statistically significant differences that *p* value ≤ 0.001.

Next, we use the two-sample *t*-test method to locate the most discriminative brain regions for MDD diagnostic classification. We extract the gray matter volume, ALFF, and ReHo values of the most discriminative ROIs from all subjects in multiple sites and construct averaged maps. The *t*-test results reveal significant GMV differences between MDD patients and NCs (*p* < 0.05, FDR-corrected), including in the bilateral anterior and posterior cerebellar cortices, left middle temporal cortex, right superior temporal cortex, bilateral inferior orbitofrontal cortex, superior and inferior frontal cortices, left hippocampus, left lingual cortex, left insula, bilateral precentral cortex, right angular gyrus, and left superior motor area. Furthermore, the ReHo values are significantly decreased in the left hippocampus, left amygdala, bilateral superior frontal cortex, left middle cingulate cortex, SMA, and inferior parietal cortex. ALFF reductions are prominent in the left middle and superior temporal cortices, left posterior cingulate cortex, and precuneus (all *p* < 0.05, FDR-corrected; see Table 3 and Figure 6).

These findings indicate that the GMVs are notably reduced in the superior temporal cortex, inferior temporal cortex, posterior cingulate cortex, orbital frontal cortex, superior frontal cortex, superior parietal cortex, supplementary motor area, and bilateral cerebellum posterior lobe, which might affect the cortico-limbic-cerebellar circuit in MDD patients [40,41,42]. We also find a significant decrease in the ALFF and ReHo values in these brain regions.

According to the visualization results, the most discriminative ROIs are primarily located in the frontoparietal network (FPN) and default mode network (DMN). Reductions in GMV and functional activity within these networks may impair their efficiency, thereby affecting perceptual switching and cognitive function in MDD patients. Furthermore, the supplementary motor area is considered the necessary cortical area for voluntary movement and also participates in cognitive activity [43]. Alterations of the SMA may be implicated in psychomotor retardation, which is a key feature in MDD patients [44]. Additionally, changes were observed in other regions critical for high-order cognitive and emotional functioning, such as decreased GMVs in the left occipital lobe and left parahippocampal gyrus, as well as reduced GMVs and ALFF in the precuneus.

The visualization analysis has verified the physiological interpretability of our pFL models, confirming that the proposed method not only achieves high diagnostic performance but also identifies neurobiologically meaningful biomarkers aligned with the established MDD pathophysiology.

## 4. Discussion

This study proposes the pF-GMCO algorithm for MDD diagnostic classification, offering a personalized federated learning framework that facilitates privacy-preserving, multisite collaboration based on adaptive gradient aggregation and contrastive optimization. The adaptive gradient aggregation mechanism in pF-GMCO incorporates domain-invariant features from multiple sites by evaluating data distribution similarities, offering a robust strategy for integrating global knowledge while mitigating domain shifts. Furthermore, federated gradient matching and model contrastive loss enable the models to be optimized towards site-specific personalization, without sacrificing global representational coherence. This unique algorithmic design ensures that each participant benefits from the collective training process while preserving localized optimization trajectories. Then, multimodal MRI data are integrated via compact bilinear pooling, which captures complex cross-modal interactions and enhances the discriminative power of the feature representation. Our approach achieves state-of-the-art performance in MDD classification, with accuracy of 79.07% across 23 sites and 2293 subjects. Notably, the identified discriminative biomarkers align with the established MDD pathophysiology, providing not only high classification accuracy but also neurobiologically meaningful interpretation.

Although the proposed method achieves promising performance in MDD diagnostic classification, there are several limitations to this study. Firstly, to ensure a fair and efficient comparison across different federated learning algorithms, we employed the 3D-ResNet-10 architecture as the backbone for all models. Although this backbone architecture has few trainable parameters and is easy to optimize, its relatively simple structure may limit its capacity to capture and encode complex neuroimaging features. We could explore more specialized backbone architectures to capture and extract features.

Secondly, to mitigate the effects of the extremely limited scale of samples in many real-world medical sites, several sites are collected into larger clients. While aggregating clients may improve the local training stability, it may introduce intraclient heterogeneity and impair the local optimization for personalized data distribution. It is necessary to pay more attention to reducing the heterogeneity within aggregated clients.

Thirdly, although the ALFF and ReHo features contributed to improving MDD classification, models trained solely on these fMRI-derived measures still underperform and remain below clinical application thresholds. ALFF and ReHo data may result in the loss of fine-grained temporal information about dynamic functional activity in 4D rs-fMRI data, which could constrain the discriminative power of the resulting features. Moreover, the rs-fMRI data used in this study were acquired at non-standardized times across sites. Since diurnal rhythms affect neuroimaging metrics [45], this temporal heterogeneity may add variability to ALFF and ReHo features. Nevertheless, despite these inherent limitations, ALFF and ReHo still provide valuable functional perspectives that are complementary to structural information. Future work should also record scan times and participant chronotypes and develop temporal harmonization methods for federated learning. Furthermore, as the pharmacological therapy status is a common and significant confounder in MRI-based MDD diagnosis, future studies must prioritize the detailed phenotyping of patients’ medication histories and dosages. This will enable the training of diagnostic models on more finely stratified subgroups, which is essential in disentangling the neural correlates of MDD from the effects of its treatment.

Finally, we only tested our proposed pF-GMCO method on an MDD diagnostic classification task in our study, but it also has potential in the diagnostic classification of multiple neuropsychiatric disorders, which needs to be explored in the future.

In summary, pF-GMCO provides a powerful tool for privacy-preserving, multisite multimodal MRI data analysis through the innovative combination of adaptive federation, personalized optimization, and multimodal integration. The method not only achieves superior diagnostic classification performance but also finds interpretable potential biomarkers for MDD. Our future work would benefit from collecting more independent medical sites and constructing federated learning applications in cross-site clinical scenarios, with significant potential to extend to multiple neuropsychiatric disorders’ diagnosis.

## 5. Conclusions

In this study, we propose a novel personalized federated learning algorithm for major depressive disorder diagnostic classification using multimodal MRI data from multiple sites. The proposed pF-GMCO effectively addresses challenges such as domain shifts through adaptive aggregation and a personalized optimization strategy, enabling us to train robust personalized federated learning (pFL) models without sharing raw data. The integration of multimodal compact bilinear pooling further enabled the discriminative fusion of sMRI and fMRI features, significantly enhancing the performance of MDD classification. Extensive validation on 23 medical sites and 2293 subjects demonstrates that pF-GMCO achieves state-of-the-art performance with average accuracy of 79.07%. The visualization analysis yielded interpretable biomarkers consistent with the known MDD neuropathology. Our study offers a scalable, privacy-preserving paradigm to train models using multisite MRI data effectively and contributes a reliable computer-aided diagnostic tool for objective and generalized MDD diagnosis.

## Figures and Tables

**Table 1:** The details of each client.

Client	Site Index	NCs	MDD	Total
Client 1	S1, S2, S3, S5, S6, S24	198	191	389
Client 2	S7, S8, S9, S10, S11	236	245	481
Client 3	S12, S13, S14, 15, S16, S23	166	234	400
Client 4	S17, S18, S21, S22, S25	217	273	490
Client 5	S20	251	282	533
Total		1068	1225	2293

**Table 2:** Averaged ACCs (%) when using different training algorithms (means ± vara).

Data	Algorithms	Client 1	Client 2	Client 3	Client 4	Client 5	Averaged
sMRI	Local	65.29 ± 0.28	88.61 ± 0.36	87.57 ± 0.36	80.61 ± 0.32	54.79 ± 0.39	74.55 ± 0.31
	Centralized	63.65 ± 0.26	86.51 ± 0.33	84.31 ± 0.30	76.58 ± 0.28	52.83 ± 0.19	71.98 ± 0.29
	FedAvg	63.34 ± 0.27	73.64 ± 0.52	77.38 ± 0.49	69.02 ± 0.43	45.96 ± 0.29	67.52 ± 0.37
	FedProx	65.46 ± 0.33	77.13 ± 0.47	79.61 ± 0.37	67.57 ± 0.42	47.39 ± 0.30	69.21 ± 0.36
	FedALA	69.17 ± 0.21	**91.47 ± 0.26**	88.46 ± 0.33	79.39 ± 0.22	55.94 ± 0.30	76.03 ± 0.23
	**pF-GMCO**	**70.95 ± 0.18**	90.45 ± 0.28	**88.76 ± 0.33**	**83.27 ± 0.26**	**57.04 ± 0.24**	**77.19 ± 0.25**
ALFF	Local	59.28 ± 0.43	67.38 ± 0.37	57.70 ± 0.46	60.20 ± 0.42	52.33 ± 0.29	58.23 ± 0.39
	Centralized	62.49 ± 0.29	63.67 ± 0.19	**61.18 ± 0.33**	55.46 ± 0.31	51.46 ± 0.19	57.49 ± 0.30
	FedAvg	47.08 ± 0.76	49.28 ± 0.94	52.13 ± 0.87	53.46 ± 0.73	47.39 ± 0.57	49.04 ± 0.78
	FedProx	51.39 ± 0.76	53.28 ± 0.72	79.61 ± 0.66	**67.57 ± 0.70**	47.39 ± 0.59	52.32 ± 0.69
	FedALA	55.77 ± 0.29	64.46 ± 0.36	65.39 ± 0.32	60.61 ± 0.35	52.72 ± 0.21	59.43 ± 0.29
	**pF-GMCO**	**62.78 ± 0.21**	**67.49 ± 0.29**	61.02 ± 0.33	62.26 ± 0.39	**53.59 ± 0.22**	**61.43 ± 0.29**
ReHo	Local	53.48 ± 0.39	63.61 ± 0.45	61.53 ± 0.43	64.69 ± 0.36	56.64 ± 0.23	60.10 ± 0.39
	Centralized	51.94 ± 0.22	62.91 ± 0.20	61.90 ± 0.29	59.81 ± 0.36	55.51 ± 0.25	58.39 ± 0.27
	FedAvg	51.39 ± 0.36	53.28 ± 0.36	69.61 ± 0.43	63.57 ± 0.50	47.37 ± 0.29	54.53 ± 0.35
	FedProx	52.02 ± 0.41	65.49 ± 0.36	63.63 ± 0.54	58.78 ± 0.63	51.89 ± 0.49	58.33 ± 0.32
	FedALA	53.96 ± 0.21	64.08 ± 0.29	66.32 ± 0.37	63.63 ± 0.28	58.16 ± 0.22	61.14 ± 0.30
	**pF-GMCO**	**54.26 ± 0.37**	**66.29 ± 0.33**	**71.25 ± 0.46**	**67.72 ± 0.31**	**61.06 ± 0.31**	**64.12 ± 0.35**
Fused MRI	Local	69.17 ± 0.21	**91.46 ± 0.26**	88.76 ± 0.33	79.39 ± 0.22	55.94 ± 0.30	76.03 ± 0.23
	Centralized	65.29 ± 0.28	88.61 ± 0.36	87.57 ± 0.36	80.61 ± 0.32	54.79 ± 0.25	74.55 ± 0.31
	FedAvg	66.29 ± 0.19	79.26 ± 0.21	78.30 ± 0.26	70.15 ± 0.29	49.27 ± 0.27	68.85 ± 0.25
	FedProx	69.62 ± 0.24	89.19 ± 0.31	88.45 ± 0.38	79.18 ± 0.35	51.60 ± 0.26	74.48 ± 0.34
	FedALA	70.05 ± 0.25	89.67 ± 0.28	88.27 ± 0.33	81.29 ± 0.29	53.26 ± 0.22	76.51 ± 0.27
	**pF-GMCO**	**71.06 ± 0.31**	91.29 ± 0.24	**89.34 ± 0.30**	**85.19 ± 0.27**	**58.45 ± 0.35**	**79.07 ± 0.29**

**Table 3:** The averaged ACCs (%) in the ablation study.

Adaptive Aggregation	$L_{\mathrm{Fed}-\mathrm{GM}}$	$L_{\mathrm{con}}$	Client #1	Client #2	Client #3	Client #4	Client #5	Averaged
✓			70.29 ± 0.21	86.28 ± 0.34	84.06 ± 0.42	79.89 ± 0.35	53.15 ± 0.28	74.73 ± 0.32
✓	✓		69.52 ± 0.23	87.26 ± 0.22	86.56 ± 0.37	82.23 ± 0.21	54.45 ± 0.29	76.00 ± 0.26
	✓	✓	68.37 ± 0.25	89.25 ± 0.23	85.00 ± 0.39	81.06 ± 0.20	54.16 ± 0.33	75.57 ± 0.28
✓		✓	69.39 ± 0.17	88.52 ± 0.35	85.27 ± 0.29	81.89 ± 0.27	56.39 ± 0.19	76.29 ± 0.25
✓	✓	✓	70.95 ± 0.18	90.45 ± 0.28	88.76 ± 0.33	83.27 ± 0.26	57.04 ± 0.24	77.19 ± 0.25

## Data Availability

The data were obtained directly from a publicly available dataset, called Data Sharing of the REST-meta-MDD Project from the DIRECT Consortium http://rfmri.org/REST-meta-MDD, (accessed on 1 January 2020), which entered the unrestricted sharing phase in 1 January 2020.
